# Goodbye or Identify: Detrimental Effects of Downsizing on Identification and Survivor Performance

**DOI:** 10.3389/fpsyg.2016.00771

**Published:** 2016-05-25

**Authors:** Rolf van Dick, Frank Drzensky, Matthias Heinz

**Affiliations:** ^1^Department of Psychology and Center for Leadership and Behavior in Organizations, Goethe UniversityFrankfurt, Germany; ^2^Work Research InstituteOslo, Norway; ^3^University of FreiburgFreiburg, Germany; ^4^Department of Economics, University of CologneCologne, Germany

**Keywords:** downsizing, organizational identification, laboratory experiment, survivor syndrome

## Abstract

Research shows that after layoffs, employees often report decreased commitment and performance which has been coined the survivor syndrome. However, the mechanisms underlying this effect remain underexplored. The purpose of the paper is to show that reduced organizational identification can serve as an explanation for the survivor syndrome. We conducted a laboratory experiment, in which participants work as a group of employees for another participant who acts as employer. In the course of the experiment, the employer decides whether one of his or her employees should be laid off or not. Mediation analysis supports a social identity-based explanation for the emergence of the survivor syndrome: downsizing causes lower identification with the employer which in turn relates to lower performance of employees.

## Introduction

Downsizing is a conventional management practice in modern economies. Between 2003 and 2011 the number of laid off US employees amounts to the size of Dallas, Texas (i.e., over 1.2 million) – every year (U.S. Bureau of Labor Statistics, 2012). Despite its wide spread usage, there is little evidence for the effectiveness of downsizing: studies find both negative and positive relationships between downsizing and firm performance or stock market reactions, respectively. However, negative effects are more likely to be the rule than the exception (e.g., Cascio et al., 1997; Nixon et al., 2004; Guthrie and Datta, 2008; for an overview see Datta et al., 2010). There are several explanations for these negative effects such as increased stress due to higher workload of the remaining employees. The effect may also be due to a lower commitment of the remaining employees – the so called survivor syndrome. In this paper, we will test this latter explanation. More specifically, we will test the causal effects of downsizing on employee identification and its effect on performance in turn using a controlled experimental design to rule out alternative explanations.

Evidence for the existence of the survivor syndrome comes from a number of field studies. This literature shows that, compared to pre-downsizing, survivors report lower job involvement (Brockner et al., 1988), commitment (Armstrong-Stassen, 1998; Allen et al., 2001), creativity (Amabile and Conti, 1999), performance (Armstrong-Stassen, 1998; Brockner et al., 2004; Travaglione and Cross, 2006), and higher feelings of insecurity and strain (De Cuyper et al., 2010). Also, absenteeism was found to be higher after downsizing (Travaglione and Cross, 2006). Although this evidence is correlative, the number of studies suggest that the survivor syndrome is a robust phenomenon.

After downsizing, remaining employees could, for instance, suffer from increased stress, guild or mistrust in the management (see Petzall et al., 2000). Given the correlative data structure, it is hard to disentangle underlying mechanisms in the field. For this reason, we seek to complement the available evidence from field studies by providing evidence from an experiment in which we studied the effects of downsizing on identification in a controlled environment. The experiment we will be presenting in this paper is based on the idea that downsizing translates into lower identification with the employer which, in turn, will explain detrimental behavior patterns of surviving employees.

To the best of our knowledge, we are the first who provide evidence that lower organizational identification might explain the behavior of surviving employees. In contrast to previous experiments, we assigned participants to the role of employers who could voluntarily decide whether to lay off an employee or not. As employers are responsible for downsizing in our experiment, we are confident to create somewhat realistic reactions of employees in response to their employer’s decisions – albeit in a laboratory setting.

Although there are a number of studies on mergers and organizational change processes, downsizing received almost no attention in the social identity literature. However, there are downsizing studies which focus on the related concept of organizational commitment (for an overview, see van Dierendonck and Jacobs, 2012). Lee and Corbett (2006), for instance, compared employees of two Korean banks after downsizing. Employees of bank A were survivors of a relatively mild downsizing (from 13 to 10 thousand employees) whereas employees of bank B were survivors of a severe program involving a reduction from 8 to about 4 thousand employees and an additional pay cut. In line with the hypotheses, the authors found that employees of bank B reported much lower affective organizational commitment and that parts of the effect were directly related to the downsizing but that the major share of the effect was due to indirect effects of the downsizing on daily work practices and experiences such as promotional chances, openness to new ideas or job complexity. To the best of our knowledge, there is no study that integrates effects of downsizing on identification and effects of identification on organizational or individual outcomes into one empirical model. Taken together, with our experimental mediation analysis, we address crucial research gaps in both, the social identity and the downsizing literature. This is important for two reasons: Establishing the causal link between downsizing and employee identification in an experimental study is theoretically important as confirmation of the theory and goes above and beyond establishing a simple relationship. Practically, it also is important to demonstrate that downsizing may negatively affect employee identification as it helps managers and change agents realizing that it is identity-related concerns of their employees that may render negative effects for their future productivity – and not (only) concerns about financial or other implications of the downsizing process.

### Theoretical Background

The basic idea of the Social Identity Theory is that people do not only think and act as individuals, but also as members of social groups. According to Tajfel and Turner (1979), social identification, i.e., the internalization of group memberships, serves the purpose of enhancing the individual’s self-esteem. The self-enhancement is evident in case of high status groups, or in the terms of Social Identity Theory, when the social identity is positive. However, other motives such as the need for predictability (Hogg and Terry, 2000) also encourage individuals to categorize themselves as members of a particular group.

Identification processes are highly relevant for organizational life. Ashforth and Mael (1989) were among the first who have introduced the concept to the organizational psychology and management field and have argued that organizational identification – in contrast to organizational commitment – strengthens the self-referential aspect of identity which helps the employees answer the question of who they are in terms of their social identity, i.e., the self concept derived from their membership in the organization. Mael and Ashforth (1992) have studied organizational identification empirically and have developed a scale to measure the construct. They found that college alumni who perceived their college more different from similar colleges and perceived it having high prestige were more likely to identify with it which in turn related to a greater willingness to recommend it and to donate money to its activities. Akerlof and Kranton (2000, p. 717) state that “…because identification is fundamental to behavior, choice of identity may be the most important ‘economic’ decision people make.” Indeed, meta-analytic results reveal positive relationships between organizational identification and, for example, job involvement, in-role performance, extra-role performance, and organizational prestige, as well as negative relationships to turnover intentions (Riketta, 2005). Identification can refer to different foci, such as the organization, work group or one’s career (Christ et al., 2003). Interestingly, there is a huge social identity literature in the context of mergers and change processes (for an overview, see Drzensky and van Dick, 2013). However, identification received almost no research attention in the context of downsizing. In one of the few exceptions, Armstrong-Stassen et al. (1996) find that nurses report lower identification with their hospital after downsizing. Brockner et al. (1987) focus on survivors’ identification with the victim. They find negative effects on employee performance when identification with the victim is high and the victim’s treatment is seen as unfair. Johnson et al. (1996) study two waves of downsizing in an insurance company and find positive relationships between organizational identification and work satisfaction.

Organizational identification is related to affective organizational commitment (see Allen and Meyer, 1990). While both concepts focus on the attachment of individuals to an organization, theoretically, only identification relies on self-categorization processes and, empirically, confirmatory factor analyses show the distinctiveness of both concepts (Gautam et al., 2004). Although the two constructs typically correlate to 0.65 or higher (see the meta-analysis by Riketta, 2005), we still see the remaining unshared variation in light of the theoretical distinctiveness as important. Therefore, we believe that a study on how downsizing affects (surviving) employees’ identification as needed despite some evidence from (questionnaire) studies on the downsizing-commitment link. Organizational identification is, amongst other things, influenced by the relationship with the immediate supervisor: Sluss et al. (2008) found that a high quality (LMX) relationship between supervisor and employee leads to more organizational identification and that this link is mediated by employees’ perception of more organizational support (POS). There are a number of studies which find that survivors report lower commitment after downsizing (e.g., Armstrong-Stassen, 1998; Allen et al., 2001; Travaglione and Cross, 2006). It was also found that, following downsizing, changes in commitment predict employee turnover (Spreitzer and Mishra, 2002). However, most studies do not focus on downsizing versus non-downsizing, but study, for instance, how fairness or justice of the downsizing process predicts changes in commitment (see van Dierendonck and Jacobs, 2012). To the best of our knowledge, there is no study which accounts for both, i.e., the effects of downsizing on identification and consequences of changes in commitment on organizational behavior.

#### Hypotheses

A number of field studies find negative associations of downsizing and (mostly self-reported) performance (Armstrong-Stassen, 1998; Brockner et al., 2004; Travaglione and Cross, 2006). However, these field studies find only weak support by previous experiments. Brockner et al. (1985, 1986, 1987, 1993) implemented an experimental design, in which two persons (the participant and a confederate) had to perform a proof-reading task. In the downsizing treatment, the confederate was laid off because of “room scheduling problems.” Only in the case of low victim’s compensation and high identification with the victim, Brockner et al. (1987) find negative effects of downsizing on the remaining employees’ performance. However, in the remaining studies (Brockner et al., 1985, 1986, 1993), survivors even increased their performance after a layoff. Note that in these experiments downsizing was implemented due to outside conditions (room scheduling). In our experiment, one participant in the role of an “employer” has to decide whether or not to lay off one of the other participants acting as “employees”. As we assume this treatment to be closer to organizational decisions, we expect to replicate evidence from the field.

From a social identity perspective, high identification is associated with embracing and following group norms. In work contexts, this typically involves high performance. Strongly identified employees should, theoretically, also support any change in the organization – such as a downsizing program – more than less identified employees. This, however, is only true as long as the change is perceived to be in the organization’s best interest. In most change contexts, however, employees might not perceive that the change helps the organization becoming a better group, which would feed back into the individual employees’ self-concept. Rather, change is often followed by resistance – particularly by the highly identified who perceive the change as a threat to the organization’s core identity and thus as a threat to their own identities as organizational members (van Dijk and van Dick, 2009). Although the two constructs typically correlate to.65 or higher (see the meta-analysis by Riketta, 2005), we still see the remaining unshared variation in light of the theoretical distinctiveness as important. Therefore, we believe that a study on how downsizing affects (surviving) employees’ identification as needed despite some evidence from (questionnaire) studies on the downsizing-commitment link. This has been demonstrated by research on mergers and acquisitions over and over again (see for an overview: Giessner et al., 2011) and also for organizational change initiatives in general (Drzensky and van Dick, 2013). We therefore propose that downsizing will negatively relate to employee performance because the downsizing will be perceived as a threat to the organization’s identity. This will lead to lower organizational identification and in turn a lower motivation to follow the organization’s norms and help accomplish its goals.

H1: Downsizing leads to lower performance of the surviving employees (survivor effect).

Employers who opt for layoffs might create the impression not to take care of their employees’ best interests. Working for an employer who opted for downsizing may thus lead to a negative social identity. Consequently, in the case of downsizing, employees should reduce their identification with the employer. This effect would experimentally replicate results from field studies (Armstrong-Stassen, 1998; Allen et al., 2001; Travaglione and Cross, 2006).

H2: Downsizing negatively relates to employees’ identification with the employer.

Given the positive relationship between identification and performance (Riketta, 2005; Riketta and van Dick, 2005), we hypothesize that reduced identification with the employer will explain the survivor effect.

H3: The survivor effect (i.e., lower employee performance post downsizing) is mediated by identification with the employer. More specifically, downsizing is negatively related to survivors’ identification with the employer, which in turn is positively related to survivors’ performance.

## Materials and Methods

### Participants and Design

Overall, 80 individuals participated in three laboratory experimental sessions – 20 in the role of employers and 60 as employees. Fifty-two percent of the participants were male, average age was 24.54 years (*SD* = 6.35). Participants were students of 18 different majors (including 14% economics students). An ethics approval was not deemed necessary for the study. The study design and all steps followed the standard economic procedure. All participants were fully aware of all the details of the study at any time. They were not deceived, were paid in real money and they knew that they could quit their participation at any time. Participation was completely anonymous – no personal information such as names were obtained at any stage of the experiment. Participants were not put under any stress at any time. We invited participants from the department of economics laboratory subject pool which comprises about 3500 students of all different majors of the Goethe-University Frankfurt using the ORSEE software (Greiner, 2004) which randomly selects and invites participants from the pool. Participants could earn real money (roughly 10–15) depending on their performance in the tasks but received no other compensation (such as course credits).

The basic design of our experiment is as follows (see also Drzensky and Heinz, 2015): For two 10 min periods, employees work on a real-effort task. Between the periods, the employer decides either to downsize or not.

By drawing a lot, we randomly divided the sample into employees and employers, respectively. Three employees were assigned to one employer. All employees were located at an individual computer workplace in the laboratory. Employers were seated in a separate room. The setting was completely anonymous and communication within or between groups was not possible. After the allocation, we distributed the instructions (first period instructions included no reference to downsizing). Participants’ task was to arrange sliders on a computer screen (see details below). We explained that the experiment would consist of two parts, but that behavior in the first part had no consequences for the second part. Employees were compensated by a fixed wage of 8€, for each period they worked for “their” employer. By earning 0.03€, for every correctly arranged slider, employers’ compensation depended on the performance of “their” employees. After period one, participants received instructions for the second period. At this point, every employer had to decide whether to lay off one employee or not. If he or she decided against downsizing, period two was identical to period one. In the case of downsizing, he or she received a fixed compensation of 5€, as an incentive to downsize plus 0.03€, for each slider correctly processed by the two surviving employees. The amount of the fixed compensation was calibrated by running a pretest in which we found that 5€, incite a sufficient number of employers to decide for downsizing.

Surviving employees earned 8€, again, while employees who had been laid off (referred to as victims in the following) received no further payment, but remained in the room until the end of the experiment. They could arrange sliders, but their effort had no consequences for themselves or the employer. To summarize, employers decided *if* they wanted to lay off one employee. Nevertheless, as the particular victim was determined by throwing dice, they could not choose *who* was laid off.

Employees were aware of their employer’s trade-off and knew that we did not reveal their first period performance. Employers, on the other hand, knew that employees were aware of their trade-off. To give employers a sense of how many sliders employees might arrange in a 10 min interval, we conducted a pretest 3 weeks before the main experiment. However, the data was provided to employers only.

### Measures

#### Performance

The slider task (see Gill and Prowse, 2012) is a computer based real-effort task. Each slider is a small button, which participants can move on a bar by using the computer mouse (arrow keys were masked). Initially, sliders are situated at the left side of the bar. A slider is successfully arranged when the button is exactly at the middle of the bar. Every second minute, a new screen with 48 sliders in three columns was presented. Thus, ceiling effects were not possible. During both working periods, employees could alternatively surf in the internet. The performance measure is the sum of correctly arranged sliders by a particular employee, separately for period one and two. In the regression analyses, we controlled for period one performance. For testing our hypotheses, we regressed performance in period two on performance in period one and estimated effects by using the residuals (which are, by definition, uncorrelated with period one performance).

#### Identification

Identification was measured with a four-item scale (Doosje et al., 1995) with the endpoints “don’t agree at all” to “completely agree.” After both periods, we asked employees about their identification with the employer (e.g. “the employer and I belong to the same group”; *α* = 0.70 – 0.71) and with the group of employees (e.g. “I feel a strong attachment to members of the group of employees”; *α* = 0.65 – 0.77).

#### Downsizing

Employers made their decision for or against downsizing by filling in a form. Thus, downsizing is a binary variable, coded 1 if downsizing was executed and coded 0 in the case of no downsizing.

## Results

**Table 1** shows the means and standard deviations separately for survivors and employees in the non-downsizing group as well as reliability coefficients and intercorrelations for both groups. As victims were excluded in period two measures (by definition, as they were laid off), for the purpose of comparability, we omitted also their period one data.

**Table 1:** Employee measures: means, standard deviations, reliability coefficients and scale intercorrelations.

	*M*_S_^a^	*SD*_S_^a^	*M*_n-d_^b^	*SD*_n-d_^b^	1	2	3	4	5	6	7	8
(1) Downsizing^c^	1	–	0	–	(–)							
(2) Identification w. employer (P1)	2.88	1.09	2.59	1.32	0.12	(0.71)						
(3) Identification w. employer (P2)	2.40	1.16	3.63	1.29	-0.45^∗∗^	0.54^∗∗^	(0.70)					
(4) Identification w. employer (P2 controlled for P1)	-0.72	0.75	0.69	1.03	-0.63^∗∗^	0.00	0.84^∗∗^	(–)				
(5) Performance (P1)	70.79	35.62	64.67	37.46	0.09	0.28^+^	0.23	0.10	(–)			
(6) Performance (P2)	44.67	53.73	77.50	36.74	-0.34^∗^	0.17	0.46^∗∗^	0.44^∗∗^	0.46^∗∗^	(–)		
(7) Performance (P2 controlled for P1)	-18.31	47.59	18.31	28.41	-0.43^∗∗^	0.05	0.39^∗∗^	0.45^∗∗^	0.00	0.89^∗∗^	(–)	
(8) Identification w. employees (P1)	3.75	1.04	3.66	1.35	0.04	0.31^∗^	0.26^+^	0.11	0.26^+^	0.11	-0.01	(0.65)

*Cronbach’s alpha reliability coefficients are shown in the diagonal; correlations and Alphas are based on the complete sample (i.e., survivors and non-downsized group); ^a^S, Survivors (N = 24), ^b^n-D, employees in non-downsized group (N = 24); ^c^Dummy variable (0 = no downsizing, 1 = downsizing); P1, before potential layoff; P2, after potential layoff; identification was measured with a seven- point scale, with 7 as highest and 1 as lowest value; performance is the number of correctly arranged sliders; ^+^p < 0.10; ^∗^p < 0.05; ^∗∗^p < 0.01*.

Since all employees were treated equally up to the beginning of period two, we should observe no difference between the groups (i.e., between survivors and those who are in a non-downsizing group) in period one. Indeed, we find no significant differences between the groups, neither for period one identification with the employer [*M*_non-downsizing_ = 2.59, *SD* = 1.32; *M*_survivors_ = 2.88, *SD* = 1.09; *t*(46) = -0.80; *p* = 0.43], nor for period one identification with the group of employees [*M*_non-downsizing_ = 3.66, *SD* = 1.35; *M*_survivors_ = 3.75, *SD* = 1.04; *t*(46) = -0.27; *p* = 0.79], nor for performance in period one [*M*_non-downsizing_ = 64.67, *SD* = 37.46; *M*_survivors_ = 70.79, *SD* = 35.62; *t*(46) = -0.58; *p* = 0.56]. This indicates that the randomization was successful.

To test Hypothesis 1 we used regression analyses. We control for identification in period one. Therefore, we regressed identification after period two on the same measure after period one and estimated effects by using the residuals. In line with Hypothesis 1, **Table 2** (column 1) shows a negative impact of downsizing on employee performance (*b* = -36.62, *p* < 0.01). That means, controlling for period one performance, employees’ performance after downsizing was about 37 sliders lower compared to the non-downsizing condition. By design, the employer’s decision for downsizing does not relate to omitted characteristics of his or her employees. As we compare period two performance between the downsizing and the non-downsizing group, also learning or fatigue cannot explain the results. Having found a survivor effect we proceed with testing identification as an explanation for the main effect.

**Table 2:** OLS regressions: hypotheses tests.

Dependent variable	Performance (P2 controlled for P1)^a^	Identification w. employer (P2 controlled for P1)^b^	Performance (P2 controlled for P1)^a^
Intercept	18.31^∗^ (8.00)	0.69^∗∗^ (0.18)	10.31 (8.97)
Downsizing^c^	-36.62^∗∗^ (11.31)	-1.42^∗∗^ (0.26)	-18.81 (14.33)
Identification w. employer (P2 controlled for P1)^b^			11.53^+^ (6.32)
*R*^2^	0.19	0.39	0.23
*N^d^*	48	47	47

*Coefficients are unstandardized regression coefficients; standard errors are in parentheses; ^a^the variable consists of the residuals of the relationship between identification with the employer in P2 and identification with the employer in P1; ^b^the variable consists of the residuals of the relationship between performance in P2 and performance in P1; ^c^binary variable (0 = no downsizing, 1 = downsizing); ^d^laid-off employees are not included, one participant did not answer the questionnaire; ^+^p < 0.10; ^∗^p < 0.05; ^∗∗^p < 0.01*.

We tested Hypotheses 2 and 3 by using the custom dialog tool PROCESS for SPSS (Hayes, 2013). The logic of the mediation model is as follows: we decompose the relationship between downsizing and performance in an indirect and a direct effect. The indirect effect (Hypothesis 3), i.e., the amount of the survivor effect that can be explained by changes in identification, is calculated by multiplying the path from downsizing to identification (first path, Hypothesis 2) with the path from identification to performance (second path). The direct effect is the (remaining) relationship between downsizing and performance when identification is controlled for.

Hypothesis 2 states that downsizing negatively affects identification with the employer. Thus, we regressed identification with the employer on downsizing (see **Table 2**, column 2). Controlling for identification in period one, we find that downsizing relates to a 1.42 point decrease in identification with the employer (*p* < 0.01). In other words, depending on the downsizing decision, identification with the employer differs by roughly one standard deviation of the initial level.

For testing both, the path from identification to performance and the direct effect, we regressed performance on downsizing and identification (see **Table 2**, column 3). We find a marginally significant relationship between identification and performance (*b* = 11.53, *p* = 0.08 two-sided; note that as our hypothesis was directed, one-sided tests resulting in significance on the 5% level could also have been applied). In line with our mediation hypothesis, the relationship between downsizing and performance is no longer significant when identification with the employer is controlled for (direct effect, *b* = -18.81, *p* = 0.20).

**FIGURE 1:**
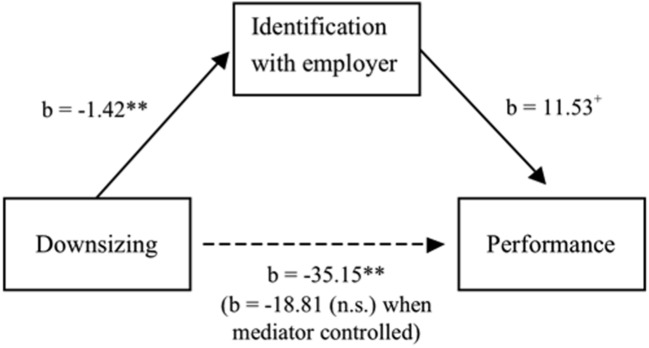
**Results of the mediation model**.

We estimated the confidence interval of the indirect effect (-1.42 × 11.53 ≈ -16.34, bias corrected 95% confidence interval = -36.54 to -2.72) by estimating 1000 bootstrap samples (see Hayes, 2013). In support of Hypothesis 3, we find that the relationship between downsizing and performance is mediated by identification with the employer (see **Figure 1**; note that the small difference in the overall relationships, *b* = -35.15 in the mediation vs. *b* = -36.62 in **Table 2**, column 1, results from missing data on identification for one participant).

To be sure that preexisting differences do not bias our results we controlled for period one measures of performance and identification. A mediation analysis without controlling for the period one variable reveals the same pattern of results – except that the overall relationship and the path from identification to performance are significant on the 5% level, the indirect effect is significant on the 1% level.

In an exploratory way, we tested whether downsizing interacts with identification with the employer or with identification with the group of employees, respectively (both in period one). We found no effects. Overall, 12 employers opted for downsizing and 8 employers kept all their employees. However, employers’ decisions cannot be predicted by their identification with the group of employees.

## Discussion

We find causal evidence for the survivor syndrome, i.e., a negative effect of downsizing on employee performance. More importantly, however, our study provides evidence that the survivor syndrome can be explained by detrimental effects of downsizing on employees’ identification with their employer.

Our results support studies indicating adverse effects of downsizing on employees’ attitudes and behavior in the field (Brockner et al., 1988; Armstrong-Stassen et al., 1996; Armstrong-Stassen, 1998; Amabile and Conti, 1999; Allen et al., 2001; Brockner et al., 2004; Travaglione and Cross, 2006) but they provide important evidence for the causal direction in a controlled environment.

Downsizing and non-downsizing groups were highly comparable and there is no common method bias. Although the experimental approach appears to be somewhat artificial, the experimental situation was real for the study participants. They were confronted with actual earnings and losses, respectively, and downsizing was based on “real” decisions without the use of cover stories or other forms of deception. As we found causal evidence in a controlled environment, our experiment strengthens also the validity of existing field studies that mostly come with the problem that they do not allow causal inferences and/or focus on either performance declines or changes in worker commitment after downsizing but do rarely combine these constructs in a single study.

Our research addresses downsizing as a research gap in the social identity literature. To the best of our knowledge, we are the first who examine both, effects of downsizing on identification and, in turn, effects of identification on performance. Thus, we are the first who provide empirical evidence for identification as a central explanation for the survivor syndrome together with evidence for the causal direction.

### Managerial Implications

Our results suggest that firms considering downsizing should account for costs resulting from detrimental behavior of survivors. Although we focused on performance, we would expect that in the field lower identification might also affect other behavior, such as OCB, creativity, deviance, or turnover intentions. These costs could be a reason why previous studies often found no positive effects of downsizing on firm performance (see Datta et al., 2010).

Effects might be especially strong if downsizing itself contradicts the firm’s identity. If, for example, workers internalize a history of non-downsizing as an integral part of their firm’s identity, downsizing will probably have a pronounced effect on identification. We assume that effects of downsizing on survivors’ identification also depend on the rationale behind and necessity of downsizing. Thus, compared to pure profit maximization, layoffs due to well-justified turnarounds may have smaller negative effects on identification.

Derived from the theoretical argument that social identification satisfies needs for esteem and predictability (Tajfel and Turner, 1979; Hogg and Terry, 2000), managers should aim to satisfy these needs to sustain employee identification – also in the context of downsizing. Thus, if downsizing processes follow a long-term strategy, managers should outline the change process as an investment in the organization’s competitiveness and efficiency, in other words, as an investment in future success and reputation (see Rousseau, 1998). Predictability might be strengthened by early and honest communication. Also participation may increase predictability and may prevent the gossip factory to become employees’ primary source of information. Good leadership might contribute to the stabilization of identification. For instance, leader-member-exchange and, consequently, organizational support were found to predict organizational identification (Sluss et al., 2008).

However, maintaining identification during downsizing seems difficult. To avoid detrimental effects on firms and employees, we recommend firms to reduce layoffs to a minimum – if they downsize at all (see for an economic cost-benefit analysis of downsizing Birati and Tziner, 2000). Firms can, for instance, make use of fluctuation instead of laying off workers or make offers for voluntary leaves. When downsizing seems to be the best or only option, managers should provide support to the employees – both to those who are leaving (e.g., by showing a genuine interest in the well-being and future and by helping them finding a new job) and who are staying (e.g., by helping them cope with the new situation which may also involve more stress). This suggestion is in line with the findings by Sluss et al. (2008) as discussed above and also with the results of Lee and Corbett (2006) who argue that perceptions of interactional justice (i.e., fair and supportive supervisor behavior) can offset the potential negative effects of employee perceptions of low distributive justice due to the downsizing. Finally, it is important that supervisors and leaders provide a sense of “projected continuity” (Ullrich et al., 2005) when communicating the downsizing. This means that leaders need to make clear why the downsizing is necessary to sustain the success of the organization in the long run which helps employees to maintain a sense of identity.

### Limitations

As groups were treated equally and showed no differences up to the beginning of period two, effects on identification and performance must be caused by downsizing. As we did not manipulate identification directly, the downside of our operationalization is that we provide only correlative evidence for the relationship between identification and performance. Nevertheless, due to the consistency with theoretical predictions and previous meta-analyses, we are confident in our interpretation of the effect.

One may also question whether it is possible to study identification processes in a short-lived laboratory experiment such as ours. First, we would like to highlight, that studying identification in group who come together for the purpose of a laboratory experiment only is common practice in psychology research (see, for instance, recent experiments by Häusser et al., 2012; Frisch et al., 2014) and that we have therefore used the scale by Doosje et al. (1995) which has been developed to measure identification in such contexts. Secondly, we want to highlight that we followed the standard economic protocol, i.e., participants came to the laboratory not to earn course credits as is the typical incentive for psychology students to participate but they participated to earn real money and were working on real tasks etc. So, we believe that the setting and manipulation for the participants resembled some reality.

In a related manner, the fact that the employer could only decide either to downsize or not but not influence which specific employee was terminated may be criticized for not reflecting organizational reality. This is certainly true for individual decisions to terminate an employee’s contract in a work group or smaller organization where the decision is (hopefully) based on that employee’s past (under)performance and thus does not signal any unfairness or callousness of the employer. In large downsizing programs of big corporations, however, this is often not the case. Here, decisions to downsize are made in some central headquarter which either closes down entire locations or departments or asks mid level management to get rid of a certain proportion of the workforce. We believe that the attributions of (surviving) employees in these cases are similar to the ones our participants make toward the employers who decided to lay off one employee – simply to safe money and irrespectively of prior performance of the employees affected by this decision.

A final limitation may apply to the generalizability of an experimental realization of downsizing and identification. Although, compared to reality, the consequences of being laid off for our participants were small (for a meta-analysis of psychological consequences of unemployment, see Paul and Moser, 2009), financial losses imposed on the victims were real. Furthermore, as indicated by a low correlation between identification and performance in the first phase, the meaning of identification seems to be low initially. However, identification became highly relevant in the second period. As in reality consequences of layoffs, social interactions and firm characteristics are much more intense, we suppose to rather have underestimated detrimental motivational effects of downsizing in this laboratory experiment.

### Future Research

Although it may be extremely difficult to find a firm (plus a control group) that would allow for a pre- and post-interventional measurement of identification and performance, it is important to replicate our mediation model in the field. For better understanding the foci of identification, future studies should test if the relationship between downsizing and performance is also mediated by organizational identification. Also identification with the group of employees received only little research attention in the context of downsizing. As we find no effects of identification with the employees, whereas Brockner et al. (1987) do, future research should discover conditions under which identification with the employees becomes relevant.

Finally, Kreiner and Ashforth (2004) have developed an expanded model of organizational identification which complements identification with the four problematic forms of disidentification, ambivalent identification, and neutral identification. In our study, we have only measured identification with the employer, but future research may also look into the effects downsizing might have on the other forms. One item of Kreiner and Ashforth’s (2004) scale to measure disidentification, for instance, asks employees to indicate the degree to which the organization “does shameful things” – one can expect that downsizing, and particularly a downsizing program that is perceived as unfair and unnecessary will be considered as such a shameful thing and disidentification might go up after such a program. Similarly, the downsizing might also lead employees to become more ambiguous about the membership in such an organization which would increase ambivalent identification.

## Conclusion

Despite the limitations discussed above, we believe that our study can be a starting point for more research in this area that focuses on identity processes – which have been largely ignored in the past. The fact that our experimental design provides causal evidence for the negative effects of downsizing on employees’ sense of identity is an important contribution. It is a step for applying this causal model to applied research exploring the link in less artificial but on the other hand somewhat “weaker” research designs such as cross-sectional surveys after the downsizing. It is also important, however, for organizational practice because it helps managers understand the negative side effects of programs designed to reduce costs but in the end risking increased costs because of adverse employee reactions. Finally, we believe that the findings of our study can enrich social identity theorizing in organizational contexts. We have emphasized in the introduction, that social identification typically is associated with following the group’s norms and aims – which, in organizations should typically translate into performance. The norms could be very different in different contexts, however, and it would be interesting to develop hypotheses about the results of similar negative effects of downsizing for employees with multiple identifications (e.g. toward organization and unions) or in service contexts where the employee may not so easily reduce his or her performance as in our experiment (which reflects more assembly line types of work).

## Author Contributions

RvD helped design the study, provided feedback on the analyses and results. FD helped design the study, collected and analyzed the data and wrote the manuscript. MH helped design the study, helped collecting and analyzing the data and provided feedback on the manuscript.

## Conflict of Interest Statement

The authors declare that the research was conducted in the absence of any commercial or financial relationships that could be construed as a potential conflict of interest.
